# A Single Dose of Azithromycin Does Not Improve Clinical Outcomes of Children Hospitalised with Bronchiolitis: A Randomised, Placebo-Controlled Trial

**DOI:** 10.1371/journal.pone.0074316

**Published:** 2013-09-25

**Authors:** Gabrielle B. McCallum, Peter S. Morris, Mark D. Chatfield, Carolyn Maclennan, Andrew V. White, Theo P. Sloots, Ian M. Mackay, Anne B. Chang

**Affiliations:** 1 Child Health Division, Menzies School of Health Research, Charles Darwin University, Darwin, Northern Territory, Australia; 2 Department of Paediatrics, Royal Darwin Hospital, Darwin, Northern Territory, Australia; 3 Department of Paediatrics, The Townsville Hospital, Townsville, Queensland, Australia; 4 Queensland Paediatric Infectious Diseases Laboratory, Sir Albert Sakzewski Virus Research Centre, Queensland Children's Medical Research Institute, Children's Health Queensland Hospital and Health Service, The University of Queensland, Brisbane, Australia; 5 Queensland Children's Respiratory Centre, Royal Children's Hospital, Brisbane, Queensland, Queensland University of Technology, Kelvin Grove, Australia; National Hospital of Utano, Japan

## Abstract

**Objective:**

Bronchiolitis, one of the most common reasons for hospitalisation in young children, is particularly problematic in Indigenous children. Macrolides may be beneficial in settings where children have high rates of nasopharyngeal bacterial carriage and frequent prolonged illness. The aim of our double-blind placebo-controlled randomised trial was to determine if a large single dose of azithromycin (compared to placebo) reduced length of stay (LOS), duration of oxygen (O_2_) and respiratory readmissions within 6 months of children hospitalised with bronchiolitis. We also determined the effect of azithromycin on nasopharyngeal microbiology.

**Methods:**

Children aged ≤18 months were randomised to receive a single large dose (30 mg/kg) of either azithromycin or placebo within 24 hrs of hospitalisation. Nasopharyngeal swabs were collected at baseline and 48hrs later. Primary endpoints (LOS, O_2_) were monitored every 12 hrs. Hospitalised respiratory readmissions 6-months post discharge was collected.

**Results:**

97 children were randomised (n = 50 azithromycin, n = 47 placebo). Median LOS was similar in both groups; azithromycin = 54 hours, placebo = 58 hours (difference between groups of 4 hours 95%CI -8, 13, p = 0.6). O_2_ requirement was not significantly different between groups; Azithromycin = 35 hrs; placebo = 42 hrs (difference 7 hours, 95%CI -9, 13, p = 0.7). Number of children re-hospitalised was similar 10 per group (OR = 0.9, 95%CI 0.3, 2, p = 0.8). At least one virus was detected in 74% of children. The azithromycin group had reduced nasopharyngeal bacterial carriage (p = 0.01) but no difference in viral detection at 48 hours.

**Conclusion:**

Although a single dose of azithromycin reduces carriage of bacteria, it is unlikely to be beneficial in reducing LOS, duration of O_2_ requirement or readmissions in children hospitalised with bronchiolitis. It remains uncertain if an earlier and/or longer duration of azithromycin improves clinical and microbiological outcomes for children. The trial was registered with the Australian and New Zealand Clinical Trials Register. Clinical trials number: ACTRN12608000150347. http://www.anzctr.org.au/TrialSearch.aspx.

## Introduction

Worldwide, bronchiolitis remains one of the most common reasons for hospitalisation of children [1]. Over 3 million children are diagnosed with bronchiolitis annually [2], [3]. The incidence of bronchiolitis is higher in some populations, including Alaskan Native and Indigenous Northern Territory (NT) infants [1]. In the latter group, hospitalisation rates for bronchiolitis are higher [4] (352 vs. 62.6 per 1000) and infections are more severe than non-Indigenous children [5].

Bronchiolitis is a clinical syndrome that is diagnosed in children up to 24 months of age [6]–[8]. The most common infecting virus, respiratory syncytial virus (RSV) occurs in 50–80% of cases, [9] although an increasing number of viruses (e.g. human rhinoviruses (HRV), coronaviruses, bocavirus), [10], [11] including multiple infections [12] are being identified.

**Table 1:** Demographic and clinical characteristics of 96 patients randomized to treatment of Azithromycin (n = 50) or placebo (n = 46) and by ethnicity.

	Azithromycin (n = 50)			Placebo (n = 46)		
	Indigenous (31)	Non Indigenous (19)	TOTAL (50)	Indigenous (30)	Non Indigenous (16)	TOTAL (46)
Age in months	5 (3–8)	5.6 (1.5–11)	5.3 (3–9.4)	5.5 (3.1–8.5)	5 (2.3–8.5)	5 (3–8.5)
Age ≤6 months	19 (61%)	9 (47.3%)	28 (56%)	18 (60%)	9 (53%)	27 (59%)
Boys	23 (74%)	11 (58%)	34 (68%)	19 (63%)	12 (75%)	31 (67%)
Gestational age (weeks)	39 (35–40)	38.3 (37–40)	39 (36–40)	38 (36–39.1)	39.2 (38.1–40)	38 (36–40)
Birth weight (kg)	3.15 (1.9–3.4)	3.38 (2.87–3.78)	3.1 (2.5–3.6)	2.82 (2.32–3.0)	3.36 (2.72–3.46)	2.87 (36–40)
Number from remote areas	19 (61%)	4 (21%)	23 (46%)	26 (87%)	3 (19%)	29 (63%)
Currently breastfed	11 (35%)	13 (68%)	24 (48%)	7 (23%)	7 (44%)	14 (63%)
Mother smoked during pregnancy	20 (65%)	2 (11%)	22 (44%)	17 (57%)	3 (19%)	20 (43.5%)
Exposed to household smoke	20 (65%)	3 (16%)	23 (46%)	24 (80%)	5 (31%)	29 (63%)
Symptoms present upon admission						
Nasal discharge	27 (87%)	16 (84%)	43 (86%)	23 (77%)	13 (81%)	36 (78%)
Cough	31 (100%)	19 (100%)	50 (100%)	30 (100%)	16 (100%)	46 (100%)
Breathing difficulties	31 (100%)	19 (100%)	50 (100%)	29 (97%)	16 (94%)	44 (96%)
Poor feeding	15 (48%)	15 (79%)	30 (60%)	10 (33%)	16 (100%)	26 (57%)
Lethargy	16 (52%)	11 (58%)	27 (54%)	19 (63%)	11 (69%)	30 (65%)
Fever °C	37 (36.3–37.2)	37 (36–37)	37 (36.2–37.1)	37 (36.3–37)	37 (36.3–38)	37 (36.3–37.1)
Antibiotics prescribed	27 (87%)	9 (47%)	36 (72%)	25 (83%)	7 (44%)	32 (70%)
Supplemental IV fluid administered	12 (39%)	7 (37%)	19 (38%)	12 (40%)	7 (44%)	19 (41%)
CXR taken	30 (97%)	14 (74%)	44 (54%)	27 (90%)	11 (69%)	38 (46%)
Co morbidities						
Otitis Media	7 (23%)	5 (26%)	12 (24%)	3 (10%)	1 (6%)	4 (9%)
Skin infection	8 (26%)	2 (11%)	10 (20%)	9 (30%)	1 (6%)	10 (22%)
Anaemia	7 (23%)	1 (5%	8 (16%)	2 (7%)	1 (6%)	3 (7%)
Failure to Thrive	1 (3%)	0 (%)	1 (2%)	1 (3%)	0 (%)	1 (2%)
Lobar Pneumonia/Collapse on CXR	8 (26%)	1 (5%)	9 (18%)	5 (17%)	0 (%)	5 (11%)
Other	1 (3%)	0 (0%)	1 (2%)	3 (13%)	2 (13%)	6 (13%)

**Median and IQR (25–75) for continuous variables. Actual numbers for categorical variables and percentages.**

**NB**: Missing variables described.

**Azithromycin**: Gestational age  = 3 (6%). Birth weight  = 6 (12%). Mother smoked during pregnancy  = 3 (6%). Exposure to household smoke  = 2 (4%), **Placebo**: Gestational age:  = 2 (4%). Birth weight  = 3 (6.5%).

**Indigenous children**: Gestational age  = 2 (3.3%). Birth weight  = 4 (6.5%). Mother smoked during pregnancy  = 2 (3%). Exposure to household smoke  = 1 (1.6%): **Non Indigenous children**: Gestational age:  = 3 (8.6%). Birth weight  = 5 (14.3%) Mother smoked during pregnancy  = 1 (3%). Exposure to household smoke  = 1 (3%).

Current recommended therapies in hospitalised children are limited to oxygen (O_2_), fluids and hypertonic saline nebulisation [7]. Antibiotics are rarely advocated in the management of bronchiolitis unless the illness is very severe or when a secondary bacterial infection is suspected [13]. However, semi-synthetic macrolides (e.g. azithromycin, clarithromycin) which have immuno-modulatory, and/or anti-microbial properties [14] and *in-vitro* anti-viral effects, [15] which may be beneficial in children with bronchiolitis and high nasopharyngeal carriage rates of bacteria. Three randomised placebo-controlled trials (RCTs) have been published and these RCTs used different doses and duration of macrolides to treat hospitalised bronchiolitis [16] [17], [18]. These studies also differed in affluence of settings which may reflect differences in the frequency and severity of acute respiratory infections in these populations [19]. Thus not surprisingly, results from the existing RCTs differed in the effect on reducing length of hospitalisation and O_2_ requirement. A Turkish [17] trial reported improved clinical outcomes. In comparison a European [16] and a Brazilian [18] trials showed no improvement.

Bacterial infections in children with RSV positive acute lower respiratory infections range from 3.5% to 31% [2], [20], [21]. The higher rate is more likely in less-affluent settings and/or with those with more severe disease [22]–[24]. Viral-bacterial co-infections are more likely when the upper airways are densely colonised with bacteria or during repeated infections [25]. In the NT, children have early acquisition of bacteria in the nasal space [26]. This is more likely to be similar to Turkey where high rates of pneumonia and bronchiectasis are also reported [22]. Thus, we conducted a RCT on children hospitalised with bronchiolitis. Our primary objective was to determine whether a single large dose of azithromycin (compared to placebo) reduced length of stay (LOS) and duration of O_2_ requirement in children hospitalised with moderate to severe bronchiolitis. We also determined the influence of azithromycin on the incidence of respiratory readmissions and presence of bacteria and viruses in the nasopharynx.

## Methods

### Study design

Our double-blinded, placebo-controlled, RCT was conducted at the Royal Darwin Hospital between June 2008 and December 2011 and The Townsville Hospital between October 2010 and December 2011.

### Trial registration

The trial was registered with the Australian and New Zealand Clinical Trials Register. Clinical trials number: ACTRN12608000150347.

### Ethics statement

The study was approved by the Human Research Ethics Committee of the Northern Territory Department of Health and Menzies School of Health Research (HREC 07/60) and The Townsville Health Service District Human Research Ethics Committee (HREC/10/QTHS/9). Individual written informed consent was obtained from children's parents or legal guardian.

### Participants

Children were enrolled if they were ≤18 months, admitted with a clinical diagnosis of bronchiolitis (according to standardised hospital protocols; ≤18 months, with cough and coryza, wheezing +/− crackles, respiratory distress with both tachypnoea (respiratory rate >50 beats/min) and retractions), required supplemental O_2_ and consented within 24 hrs of hospitalisation. Children were excluded if they had: severe disease (admitted to intensive care unit); chronic lung disease, congenital heart disease, contraindications to macrolide use (e.g. liver dysfunction, hypersensitivity), diarrhoea (>2 stools of watery consistency more than normal pattern), received macrolides (in last 7-days), or clinical and radiological features consistent with a primary diagnosis of pneumonia [27] at time of randomisation.

### Protocol and interventions used across both sites

Study staff visited the paediatric wards twice daily to assess newly admitted children. After consent, children were randomised to receive a single large dose of oral liquid azithromycin (30 mg/kg) or placebo suspension (equal volume). The placebo suspension was made up of confectioner's Sugar, Hydroxypropyl Cellulose, Xanthan Gum, Syloid 244, Sodium Phosphate Tribasic, Imitation Vanilla Creamy Flavour, Black Cherry Flavour, Quinine Sulphate (ground Quinate 300 mg Tablets). Children were managed by the paediatric team of each hospital according to the same clinical protocol for bronchiolitis (e.g. criteria for commencement and weaning of O_2_) that was standardised >6 months before commencement of the trial. Children were allowed to receive concurrent medications specified by the attending physician, except macrolide antibiotics. The protocol for this trial and supporting CONSORT checklist are available as supporting information; see Checklist S1 and Protocol S1.

### Randomisation, allocation and blinding

Randomisation was stratified by age (≤6 or >6 months), ethnicity (Indigenous or non-Indigenous) and site (Darwin or Townsville). Randomisation was by computer generated permuted blocks and treatment allocation concealed by opaque stickers. Upon enrolment, children were assigned the next treatment on the appropriate stratified list. Neither the study team (researchers, hospital staff) nor parents were aware of the assigned treatment group until the end of the trial.

The placebo medication was manufactured by the Institute of Drug Technology Australia Limited (Melbourne, Victoria). It had a similar smell and taste to active azithromycin. Azithromycin (Pfizer, Australia) was repackaged by IDT. Both medications were prepared as powder in identical opaque bottles and sealed with an aluminium foil.

### Clinical assessment and outcome measures

Standardised data collection forms were used to record demographic, medical history and clinical data from each child (table-1). This included O_2_ requirement and level, respiratory rate, temperature and heart rate. Other therapies (intravenous fluids, antibiotics) and routine investigations (full blood count and chest x-ray) were also documented.

**Table 2:** Subgroup analysis of LOS and time on O_2_ by ethnicity and age.

	Azithromycin	Placebo	Difference	95% CI
			(placebo-Azithromycin)	
**Length of Stay (LOS) median hours**				
**Ethnicity**				
** Indigenous**	57	61	3	(−13, 20)
** Non Indigenous**	46	54	4.5	(−11, 13)
**Age**				
** <6 months**	63	61	2.3	(−18,19)
** >6 months**	47	51	0.7	(−9, 11)
**Time on oxygen (O_2_) median hours**				
**Ethnicity**				
** Indigenous**	39	47	4	(−12, 22)
** Non Indigenous**	30	36	4	(−17, 12)
**Age**				
** <6 months**	46	43	2	(−18,19)
** >6 months**	30	32	0.7	(−9, 12)

Children enrolled in the study were assessed twice daily by the attending doctor (blinded and not an investigator) for clinical signs inconsistent with bronchiolitis and associated with known azithromycin side effects. Outcome measures were collected every 12 hours until the study endpoint was reached. The primary endpoints were: LOS for respiratory illness and duration of O_2_ requirement. LOS was defined as time from admission to time for ‘ready for discharge’ (Sp0_2_ consistently >94% in air for >16 hrs) and feeding adequately. ‘Ready for discharge’ differed from LOS, as discharge from hospital in our setting is often delayed due to other social factors, especially in children from remote Indigenous communities. Other outcomes were (i) any respiratory related readmissions within 6 months of discharge and (ii) identification of respiratory viruses and bacterial pathogens. Adverse events were monitored by study staff every 12 hours until discharge. Respiratory readmissions were collected from the medical charts; as there these children had no access to any other hospitals in the region, this is a reliable outcome.

### Specimen collection and process

A nasopharyngeal swab (NPS) was taken prior to administration of study medication and 48 hrs later (or at discharge). NPS were placed in skim milk tryptone glucose glycerol broth media and were transferred on ice stored at −80°C in accordance to published guidelines [28], [29].

Assessment for viruses and atypical bacteria were described previously [30]–[32]. Nucleic acids were extracted from 0.2 ml of each NPS using the High Pure Viral Nucleic Acid kit (Roche Diagnostics, Australia), according to the manufacturer's instructions. Polymerase Chain Reaction (PCR) methods were used to detect RSV (A and B), adenovirus, parainfluenza (1, 2, 3), influenzavirus (A and B), HRV and enterovirus, coronaviruses, bocavirus type 1, human metapneumovirus (hMPV), KI (KIPyV) and WU (WUPyV) polyomaviruses, *Chlamydophila pneumoniae* and *Mycoplasma pneumoniae*. For bacterial analysis, NPS were thawed and 10 µL aliquots cultured overnight on selective media at 37°C in 5% CO_2_; identification of *Streptococcus pneumoniae*, *Haemophilus influenzae*, *Moraxella catarrhalis* and *Staphylococcus aureus* used established techniques as previously described [26], [28], [33].

**Table 3:** Bacteria outcomes at pre treatment (baseline) and post treatment (48 hours).

	Azithromycin		Placebo		Azi vs. Placebo
	Baseline	48 hours	Baseline	48 hours	48 hours
					P value
Nasal carriage of pathogens	N = 49	N = 34	N = 46	N = 37	
* Streptococcus pneumoniae*	9 (18%)	2 (6%)	15 (33%)	7 (19%)	0.15
* Haemophilus influenzae*	18 (37%)	3 (9%)	18 (39%)	10 (27%)	0.06
* Moraxella catarrhalis*	21 (43%)	2 (6%)	16 (35%)	12 (32%)	0.006
* Staphylococcus aureus*	4 (8%)	1 (3%)	4 (9%)	1 (3%)	1.00

### Statistical methods

We formally compared baseline characteristics of Indigenous vs. non-Indigenous children, with appropriate statistical tests. We did not formally do this between treatment groups in accordance with current CONSORT recommendations (available http://www.consort-statement.org/consort-statement/13-19-results/item15_baseline-data/. Accessed 28^th^ June 2013). Our pre-specified analysis plan, stated that non parametric methods be used if data were not normally distributed. Data were presented as medians and interquartile range (IQR) for LOS and O_2._ Differences between groups were tested using the Mann-Whitney test. A 95% confidence interval (CI) was obtained for the difference in medians between treatment groups [34]. Subgroup analysis was performed by ethnicity (Indigenous vs. non-Indigenous) and by age (≤6 and >6 months). Differences in proportions were tested with Fisher's exact test. We looked at time to readmission within 6 months of hospital discharge using Kaplan-Meier survival plots.

### Sample size

We calculated that a total sample of 92 children (equal numbers of Indigenous and non-Indigenous children recruited) would provide 90% power to detect a difference in the mean LOS of 24 hrs between each treatment for each ethnic group at the 5% significance level assuming the standard deviation was 24 hrs in each group. This study was underpowered to detect differences in rates of readmission between treatment groups.

## Results

**Figure 1:**
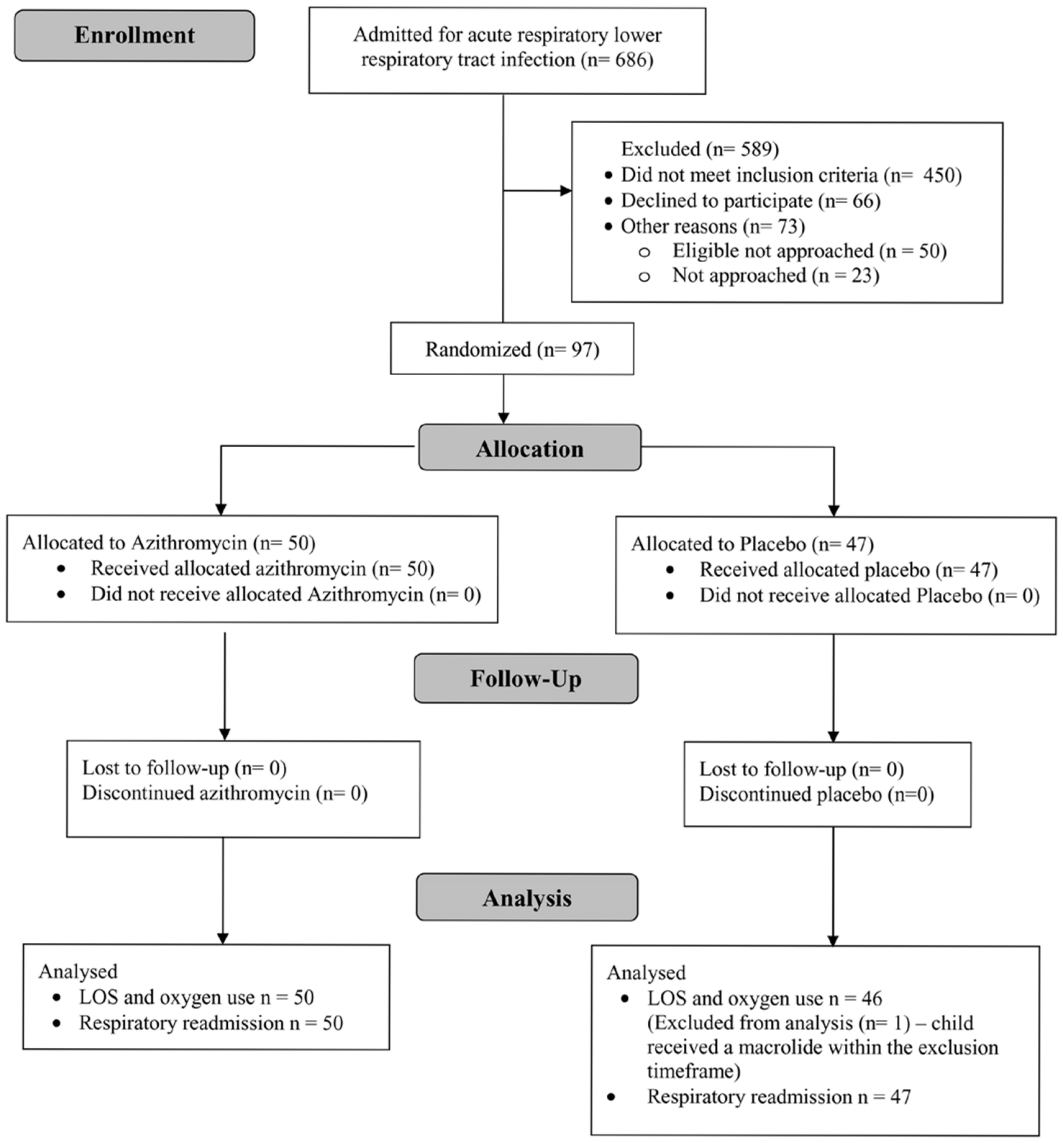
CONSORT trial overview.

We recruited 97 children and data from 96 children were analysed (Figure 1). The major reason why 450 children did not meet the inclusion criteria was they did not require supplemental O_2_ or were admitted over the weekend. During recruitment, 21 children admitted into intensive care were excluded; 17 were Indigenous. One participant was excluded from the analysis of primary outcomes; they had received a macrolide in the previous 7 days (this child was randomised to placebo). This child was included in the analysis of secondary outcomes. Of the 96 remaining children, demographic and clinical characteristics were similar between the treatment groups (Table-1). No children received steroids during hospitalisation. Of the cohort, 10 children were previously hospitalised for a respiratory episode, all were Indigenous; 3 in azithromycin group and 7 in placebo group.

When data were grouped by ethnicity, a higher proportion of Indigenous children lived in remote areas (n = 45, 74%; p = <0.001), were exposed to cigarette smoke during pregnancy (n = 37, 61%; p = <0.001), or in their household (n = 44, 72%; p = <0.001). They were more likely to have coexisting co-morbidities (i.e. skin infections (n = 17, 28%; p = 0.02) or secondary pneumonia (n = 13, 21%; p = 0.01). More Indigenous children (n = 34, 56%, p = <0.001) received antibiotics prior to hospitalisation. The antibiotics given were ceftriaxone (n = 14, 41%), procaine penicillin (n = 7, 21%) and amoxicillin (n = 5, 15%). In hospital, additional antibiotics were more often prescribed in Indigenous children (n = 52, 85%, p = <0.001) (Table-1).

**Figure 2:**
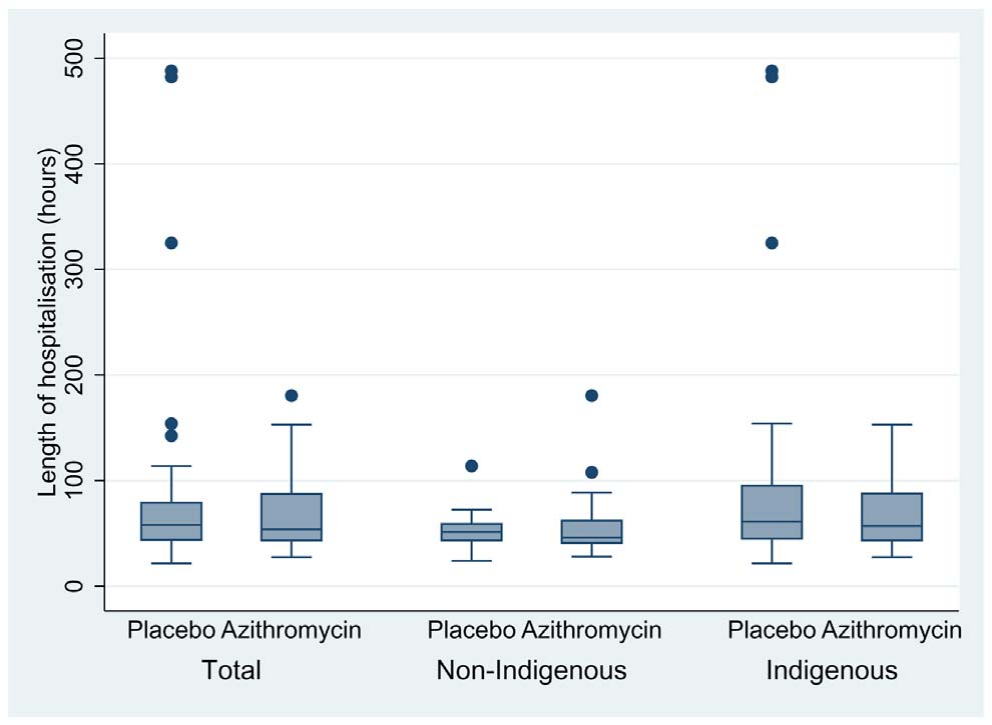
Length of stay (LOS) in hospital – Azithromycin Vs Placebo and Ethnicity.

**Figure 3:**
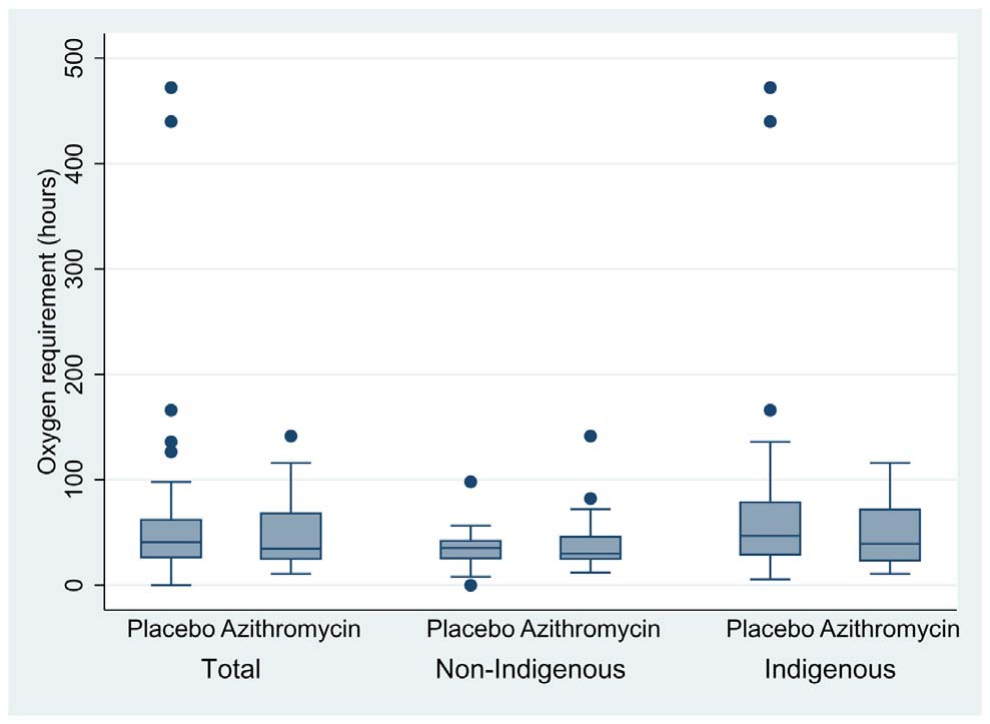
Time on Oxygen (O_2_) in hospital – Azithromycin Vs Placebo and Ethnicity.

LOS was similar in both treatment groups. The median LOS in the azithromycin group was 54 hrs, compared to 58 hrs in the placebo group (difference between groups of 4 hrs, 95%CI −8, 13, p = 0.6), figure 2. The median time on O_2_ in the azithromycin group was 35 hrs, compared to 42 hrs in the placebo group (i.e. reduction of 7 hrs 95%CI −9, 13, p = 0.7), figure 3. No child required admission into intensive care and there were no adverse or serious adverse events.

**Figure 4:**
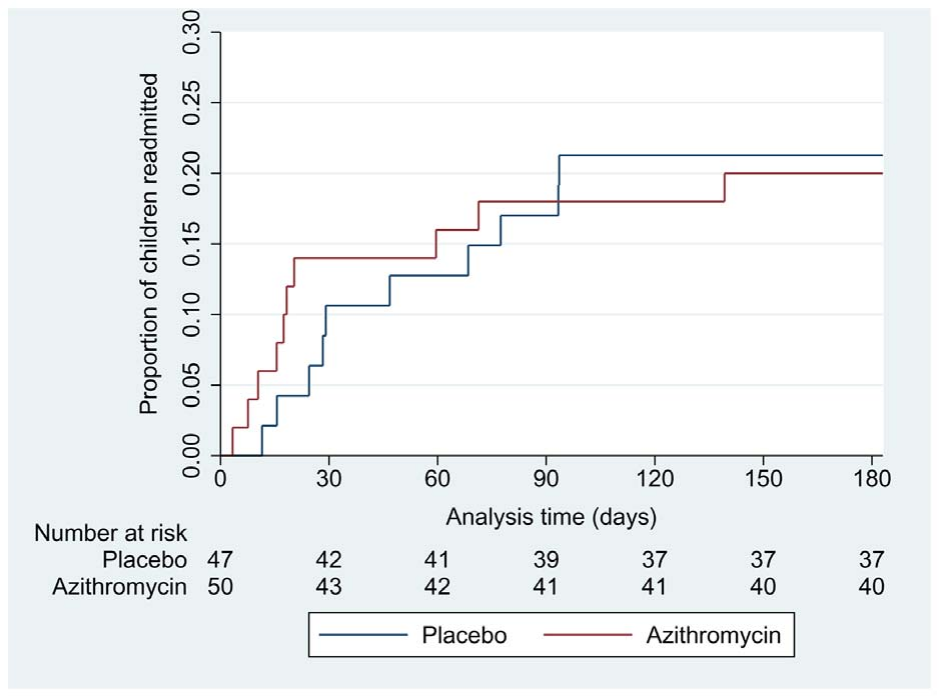
Time to first readmission – Azithromycin Vs Placebo.

All children contributed to readmission data. There was no significant difference in the number of respiratory readmissions within 6 months (10 per group, OR = 0.9, 95%CI 0.3, 2, p = 0.8) or time to readmission (logrank p = 0.9) between treatment groups (figure 4). 70% of children readmitted, were reported to have a wheeze-associated illness.

**Figure 5:**
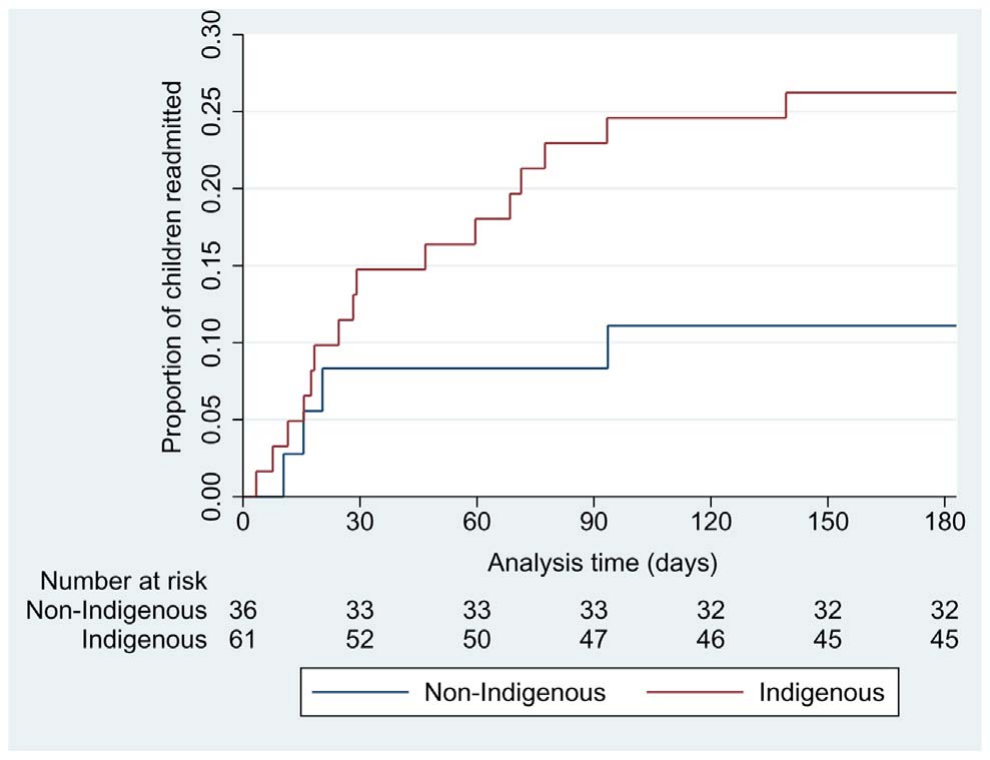
Time to first readmission – Indigenous Vs Non-Indigenous.

Indigenous children (n = 61) had longer LOS; median 59 hrs compared to 51 hrs in non-Indigenous children (n = 35) (difference of 8 hrs, 95%CI -25, 1.5, p = 0.07). This was similar with duration of O_2_; 43 hrs in Indigenous children and 35 hrs in non-Indigenous children (difference of 8 hrs 95%CI -22, 1.4, p = 0.08). A higher proportion of Indigenous children were readmitted for a respiratory illness (n = 16 (26%) compared to non-Indigenous children (n = 4 (11%)), difference 15% (95%CI 0, 30%) p = 0.05. Indigenous children were more likely to be re-hospitalised earlier (Indigenous n = 16, non-Indigenous n = 4, OR = 2.8, 95% CI 0.9, 8.8), logrank p = 0.08 (figure 5). There was no evidence that the difference in either LOS or O_2_ between treatment groups varied according to ethnicity or age (table-2).

### Viral and bacteria data

All but one child had a baseline NPS. NPS could not be obtained on all participants at 48 hrs due to discharge occurring during evenings or weekends. One participant's family withdrew consent for NPS.

**Figure 6:**
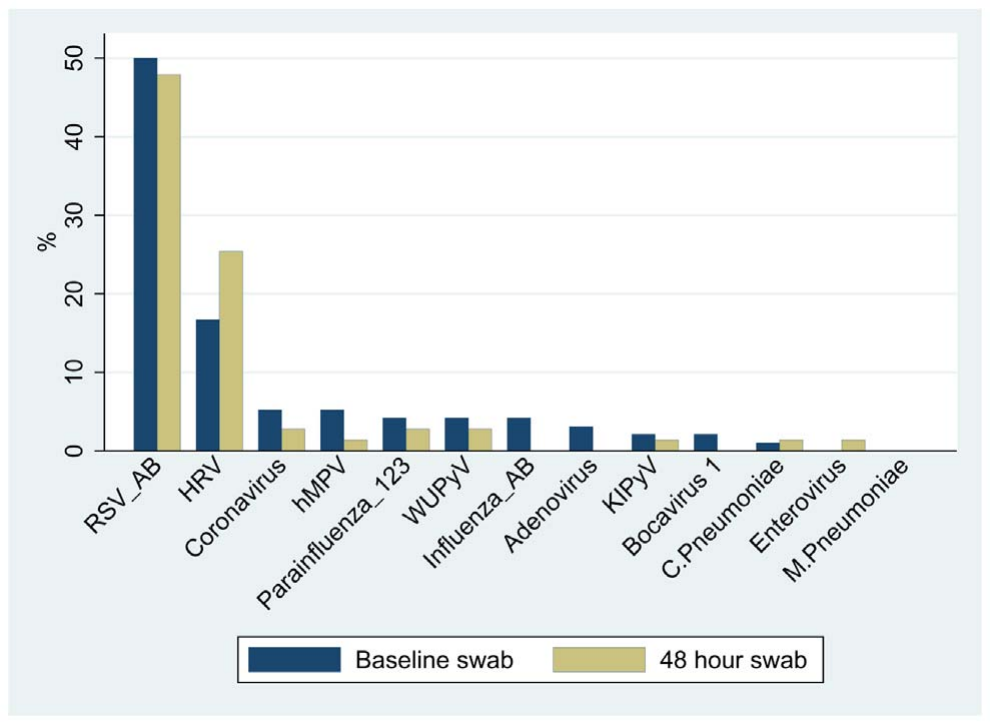
Frequency of viruses detected in NPS – Baseline and 48 hours.

At baseline, viruses were not detected in 23 (24%) participants. One or more virus was detected in 54 (56%) children. Two or more viruses were detected in 19 (20%) of children. RSV was the most common (n = 48, 50%), followed by HRV (n = 16, 17%), hMPV (n = 5, 5%) and coronavirus (n = 5, 5%). Figure 6 depicts the frequency of virus detection at baseline and 48hrs. There was no reduction in the mean number of viruses detected per child from baseline to 48hrs; azithromycin 1.0 to 0.8 (95% CI −0.2, 0.6, n = 34), placebo 0.9 to 1.0 (95% CI −0.3, 0.2, n = 37).

Table-3 summarises NPS bacteria detected at baseline and 48 hrs. A reduction in the mean number of respiratory bacteria was detected per child; in the azithromycin group from 1.2 to 0.5 bacteria (difference 0.7 95%CI 0.25, 1.1, p = 0.01), and zero change in the placebo group; 1.3 to 1.3 bacteria.

Compared to baseline NPS, children who received azithromycin alone (n = 14) (i.e. received no additional antibiotics in hospital) were less likely to have *S. pneumoniae* (3/14, vs. 0/11), *M. catarrhalis* (5/14 vs. 1/11), *H. influenzae* (6/14 vs. 1/11), and *Staphylococcus aureus* (1/14 vs.0/11) at 48 hrs. 3/14 children did not have NPS at 48 hrs.

## Discussion

We found that a large single dose of azithromycin (compared to placebo), did not have large clinical effects on LOS, length of O_2_ requirement or readmission within 6 months of discharge. Azithromycin reduced the proportion of children with respiratory bacteria in their NPS but had no significant effect on viral detection by PCR.

Of the 3 published RCTs on macrolides to treat hospitalised children with bronchiolitis, [16]–[18] only one [17] reported improved clinical effects i.e. reduced LOS, duration of O_2_ requirement and lower readmission rates. Our findings are similar to the other two trials [16], [18] showing that a single dose azithromycin does not shorten LOS and O_2_ requirement. However, methodological differences among trials need to be considered. One of the larger trials [16] included only children with RSV-confirmed bronchiolitis, thus limiting generalisation to bronchiolitis caused by other pathogens. Other differences included: age, ethnic populations, concurrent use of antibiotics, treatment practices and macrolide type, dose and duration. The immunomodulatory difference between azithromycin and clarithromycin may also account for the differential results between Tahan et al's study [17] with ours and the other 2 RCTs [16], [18]. For example, azithromycin increased the production of IL-10 whereas clarithromycin inhibited the production of IL-6 by dendritic cells in animal work [35]. Tahan and colleagues [17] used a daily dose for 3-weeks of clarithromycin, but ours like Kneyber et al [16], [18] (7-day daily dose) used a short course of azithromycin. However, Tahan et al's [17] study had very small numbers (n = 21) and high attrition. Thus, it remains unknown if a longer course of azithromycin may be effective in reducing readmission rates.

Of the published RCTs on macrolides for bronchiolitis in children, our patient profile is most like that of the Turkish study [17]. However, unlike the Turkish RCT, [17] we did not find a beneficial effect of azithromycin on clinical outcomes. Possibly contributory reasons include the very high concomitant use of antibiotics in our group; different treatment regime used the density of bacterial carriage and secondary co-morbidities. The common use of antibiotics in children with bronchiolitis in our setting relates to the high rates of concomitant infections among children. A similar treatment practice occurs in Alaskan children [1]. 56% of Indigenous children in our trial received antibiotics before admission and 87% during hospitalisation. While the use of antibiotics is common practice in such settings, its effectiveness and possibly increased adverse events remains unknown. Ideally concurrent antibiotics should have been disallowed in our study but it was not possible to alter clinicians' practice and our *a priori* protocol allowed the concurrent use of antibiotics other than macrolides.

We used a single large dose of azithromycin, which is equivalent to one week of treatment for several reasons [36]. In our setting, early (as early as 2-weeks of age) nasopharyngeal colonisation of respiratory pathogens occurs in Indigenous children [37]. Azithromycin potentially has a beneficial microbiological effect on these pathogens [36]. Azithromycin also has the benefit of a long half life and tissue penetration requiring less frequent dosing, compared to other antibiotics [36]. This is important in our setting where adherence to treatment regimes can be challenging.

While our trial did not find significant differences between treatment groups for clinical outcomes, our study had some novel data. Firstly, none of the published RCTs on macrolides for children with bronchiolitis report data on the impact on viral detection or bacterial carriage. As viruses were identified by PCR, it is not surprising no difference in viral detection were found at 48 hrs (although azithromycin may have some anti-viral effect) [15]. Future research should look at the impact of azithromycin on viral load/copies. While our numbers were small, we showed a significant difference in the mean number of respiratory bacteria per child; from 1.2 to 0.5 bacteria (difference 0.7 95%CI 0.25, 1.1, p = 0.01) in the azithromycin group. This is important in our setting as NPS carriage of respiratory pathogens (e.g. *S. pneumoniae, H. influenzae, M. catarrhalis*) are among the highest reported globally (over 80%), compared to non-Indigenous children (50%) [26].

Secondly, our study provides a clinical picture of hospitalised cases of bronchiolitis in different geographical and ethnic groups in Northern Australia, where acute respiratory infections may be one precursor of high rates of chronic respiratory illness [5], [38] This is the first data published, showing NPS detection of viruses and bacteria from Indigenous children hospitalised with bronchiolitis. Wheezing and bacterial infections in young children have been shown to be associated in one prospective cohort study [39]. Thus our data may have implications in settings, where acute and chronic respiratory diseases are particularly prevalent and more severe, including Alaska and New Zealand [40]–[42].

We found that Indigenous children exhibited longer LOS, O_2_ requirement and earlier time to hospital readmission than non-Indigenous children. The most likely reason why the latter aspects are different from our previous study [5] is because we excluded children managed in intensive care. In our cohort, Indigenous children were more likely to be readmitted for another respiratory illness. This is not surprising as Indigenous children in the NT are 5 times more likely to be hospitalised for pneumonia and influenza than non-Indigenous children [43]. Whether readmission is related to the insult from bronchiolitis can only be postulated. Recurrent hospitalisation for respiratory illness is an independent risk factor for developing bronchiectasis and/or respiratory dysfunction in adulthood [44], [45]. In our region, bronchiectasis affects 1 in every 68 Indigenous children [46]. In the follow up of our cohort, 6/61 (10%) Indigenous children (3 in azithromycin group, 3 in placebo group) have subsequently been diagnosed with bronchiectasis and an additional 4 children are awaiting chest scans.

The prevalence of readmission for a respiratory illness within 6-months in our trial was 21%; 70% had a wheezing illness. This was similar to the Turkish trial at 24% (53% were wheezing) [17]. The two most common viruses found in our cohort, RSV and HRV have been implicated for ongoing wheezing [47]–[50]. New Zealand data have also recently described high prevalence (70%) of on-going intermittent wheeze 12-months post hospitalisation with acute lower respiratory infections [41]. In addition, wheezing and persistent cough can also be problematic post acute bronchiolitis [17]. Our trial (and the other published RCTs of macrolides for acute bronchiolitis) did not assess this, a known clinical research gap [7], [51].

Despite providing new data, there are other several limitations to our study, in addition to the concurrent use of antibiotics. Having older children increased the risk of including asthma prone children. We also did not limit to the first bronchiolitis admission. Removing the children with recurrent disease in a secondary analysis made no difference to study outcomes. As our study was limited to a single dose, it remains uncertain if any macrolides, or a longer macrolide treatment course, is beneficial in high risk children who do not receive any other antibiotics. Only having two sites, may also affect the generalisability of the results.

## Conclusion

In children hospitalised with moderate to severe bronchiolitis and requiring supplemental O_2_, we found that a large single dose of azithromycin (compared to placebo) did not have any clinical benefit to reduce LOS, duration of O_2_ requirement or readmission rates within 6 months of hospital discharge. Azithromycin reduced the proportion of bacterial carriage, but had no significant effect on reducing proportion of viruses. Further research is required to determine whether earlier administration and longer duration of azithromycin is beneficial to improve the clinical and microbiological outcomes of acute bronchiolitis, associated co-morbidities and prevent ongoing respiratory morbidity in this population.

## Supporting Information

Checklist S1 **CONSORT checklist.**
(PDF)

Protocol S1 **Trial protocol.**
(PDF)
