# Supplementary material for: A Single Dose of Azithromycin Does Not Improve Clinical Outcomes of Children Hospitalised with Bronchiolitis: A Randomised, Placebo-Controlled Trial
Source: PLoS One. 2013 Sep 25;8(9):e74316. doi: 10.1371/journal.pone.0074316 (PMC3783434; doi:10.1371/journal.pone.0074316)
Supplement: Protocol S1 — Trial protocol. (PDF) [file pone.0074316.s002.pdf]

Royal Children's Hospital
Brisbane, Qld

Queensland Government
Queensland Health
Townsville Health Service District
Women's and Children's Health Institute
Department of Paediatrics

James Cook University
Australia

Queensland Children's Medical Research Institute

Northern Territory Government
DEPARTMENT OF HEALTH AND FAMILIES

An Intervention study for bronchiolitis: Improving the management and outcomes for infants hospitalised with bronchiolitis (ABIS)

Clinical study protocol – Field Version
Version 2 – 2nd September 2010

**Project Coordinator**	Gabrielle McCallum
**Site Coordinators**	Professor Anne Chang<br>Dr Carolyn Maclennan<br>Associate Professor Peter Morris<br>Dr Andrew White
**Research Nurses**	Nerida Jacobsen<br>Clare Wilson<br>Lesley Versteegh<br>Kobi Schutz<br>Susan Pizzutto
**Funding**	Channel 7 Children's Research Foundation<br>Financial Markets Foundation for Children
**Duration**	2010 - 2012
**Project code:**	R317A

menzies
school of health research
discovery for a healthy tomorrow
PO Box 41096, CASUARINA NT 0811 | John Mathews Building (Building 58), Royal Darwin Hospital Campus, Rocklands Drive, CASUARINA NT 0810 Phone: 08 8922 8196 | Facsimile: 08 8927 5187 | Web: www.menzies.edu.au | Email: info@menzies.edu.au

Chief Investigators

Dr Carolyn Maclennan
Royal Darwin Hospital
Rocklands Drive
Tiwi NT 0811

Professor Anne Chang
Menzies School of Health Research
John Mathews (Building 58)
Royal Darwin Hospital Campus
Charles Darwin University
PO Box 41096
Casuarina NT 0810

Investigators

Associate Professor Peter Morris
Menzies School of Health Research
John Mathews (Building 58)
Royal Darwin Hospital Campus
Charles Darwin University
PO Box 41096
Casuarina NT 0810

TABLE OF CONTENTS

1. **List of abbreviations**
2. **General Information**
   - 2.1. Protocol full title
   - 2.2. Trial Sponsor
   - 2.3. Person(s) authorised to sign the protocol and the protocol amendments for the sponsor
   - 2.4. Investigator responsible for conducting the trial and address and telephone numbers of the trial sites
   - 2.5. Qualified physician responsible for all trial-site related medical decisions
   - 2.6. Confidentiality statement
3. **Study Design**
   - 3.1. Hypothesis
   - 3.2. Aim
   - 3.3. Significance
   - 3.4. Background and rationale
     - 3.4.1. Why use Azithromycin for bronchiolitis
     - 3.4.2. Why study immune responses
   - 3.5. Flow chart-intervention arm
   - 3.6. Study timeline
4. **Study design**
   - 4.1. Eligibility criteria
   - 4.2. Enrolment protocol
     - 4.2.1. Enrolment procedure
     - 4.2.2. Randomisation and blinding of treatment allocation
     - 4.2.3. Clinical samples
       - 4.2.3.1. Infants with Bronchiolitis
   - 4.3. Study size
   - 4.4. Refusal to participate
   - 4.5. Withdrawal of participant
   - 4.6. Clinical assessment
   - 4.7. Microbiology/Virology
     - 4.7.1. Bacteriology of nasopharyngeal swabs
     - 4.7.2. Respiratory pathogens in nasopharyngeal aspirates

4.8. Data analysis

4.8.1. Primary outcome

4.8.2. Secondary outcome

5. Data storage and record keeping

6. Quality control/quality assurance

6.1. Safety parameters

6.2. Methods of assessing safety parameters

6.3. Flow chart – adverse event reporting

6.4. Classification and reporting serious adverse events

6.5. Data safety monitoring board

6.6. DSMB independent safety monitor

7. Publication policy

8. Ethics

9. Registering of trial

10. References

1. List of abbreviations

**AE**	Adverse Event
**CI**	Chief Investigator
**CSLD**	Chronic Suppurative Lung Disease
**CXR**	Chest X-ray
**DSMB**	Data and Safety Monitoring Board
**GCP**	Good Clinical Practice
**HREC**	Human Research Ethics Committee
**IDT**	Institute of Drug and Technology
**Menzies**	Menzies School of Health Research
**NHMRC**	National Health and Medical Research Council
**NT**	Northern Territory
**NPA**	Nasopharyngeal aspirate
**NPS**	Nasopharyngeal swab
**PBMC**	Peripheral Blood mononucleocyte
**QPIDL**	Queensland Paediatric Infectious Diseases laboratory
**RCT**	Randomized Clinical Trial
**RDH**	Royal Darwin Hospital
**SAE**	Serious Adverse Event/Serious Adverse Experience
**SOP**	Standard Operating Procedure
**TTH**	The Townsville Hospital
**WHO**	World Health Organization

2. General Information

2.1. Protocol title

An Intervention study for bronchiolitis in Darwin and Townsville: Improving the management and outcomes for infants hospitalised with bronchiolitis.

2.2. Trial Sponsor

Royal Darwin Hospital
Rocklands Drive
Tiwi NT 0811

2.3. Person(s) authorised to sign the protocol and the protocol amendments for the sponsor:

Professor Anne Chang
Menzies School of Health Research
John Mathews Building 58
Royal Darwin Hospital Campus
Charles Darwin University
PO Box 41096
Casuarina NT 0810
Ph: 61-8-8922 8371

Dr Carolyn Maclennan
Royal Darwin Hospital
Rocklands Drive
Tiwi NT 0811
Ph: 61-8-8922 8765

2.4. Investigator responsible for conducting the trial and address and telephone numbers of the trial sites

Professor Anne Chang
Menzies School of Health Research
John Mathews Building 58
Royal Darwin Hospital Campus
Charles Darwin University
PO Box 41096
Casuarina NT 0810
Ph: 61-8-8922 8371

Dr Carolyn Maclennan
Royal Darwin Hospital
Rocklands Drive
Tiwi NT 0811
Ph: 61-8-8922 8765

2.5. Qualified physician responsible for all trial-site related medical decisions

Professor Anne Chang
Menzies School of Health Research
John Mathews Building 58
Royal Darwin Hospital Campus
Charles Darwin University
PO Box 41096
Casuarina NT 0810
Ph: 61-8-8922 8829

Dr Carolyn Maclennan
Royal Darwin Hospital
Rocklands Drive
Tiwi NT 0811
Ph: 61-8-8922 8765

Dr Peter Morris
Menzies School of Health Research
John Mathews Building 58
Royal Darwin Hospital Campus
Charles Darwin University
PO Box 41096
Casuarina NT 0810
Ph: 61-8-8922 8371

Dr Andrew White
The Townsville Hospital
PO Box IMB 52
Douglas 4811 QLD
Ph: (07) 4796-1776

2.6. Confidentiality statement

All information found within is the property of Menzies School of Health Research and therefore provided to you in confidence. Authorised personnel may only access this information and it is understood that its contents shall not be disclosed without written authorisation from the Chief Investigator.

3. Study Design

3.1. Hypothesis

a) The combined anti-microbial and anti-inflammatory properties of a macrolide, azithromycin, will improve clinical outcomes for infants hospitalised with moderate to severe bronchiolitis.

b) That co-infection with other respiratory pathogens alters an infant's immune response and is responsible for the differences seen in the clinical recovery of infants with bronchiolitis.

3.2. Aim

Assess the efficacy of a single dose of Azithromycin (30mg/kg) to

a) Improve the clinical course of moderate to severe bronchiolitis (assessed by supplemented oxygen requirement and length of hospital stay).

b) reduce the risk of further respiratory illness within 6 months of this bronchiolitis episode.

c) Profile the immunopathology of bronchiolitis in the presence and absence of co-existing respiratory infections.

3.3. Significance

Bronchiolitis is the most common lower respiratory tract infection in infancy. It is primarily caused by infection of the respiratory epithelial cells by Respiratory Syncytial Virus (RSV) although other respiratory viruses including adenovirus, parainfluenza, influenza and human metapneumovirus may also contribute. Bronchiolitis is characterised by extensive inflammation of the lower airways with an associated increase in mucus production and necrosis of epithelial tissue. Clinically, bronchiolitis is manifested by presence of tachypnoea with wheeze and/or crepitations with a preceding upper respiratory illness.

3.4 Background and rationale

3.4.1 Infants with bronchiolitis in the NT: burden and outcomes

Bronchiolitis (with or without pneumonia) is the most common cause of admission in children aged under 12-months. In a Central Australian study, we described a prevalence of approximately 190 per 1000 children for RSV bronchiolitis.[1] The prevalence of all causes of bronchiolitis would indeed be higher. In our setting, co-existent pneumonia is high in Indigenous children and, at the Royal Darwin Hospital (RDH), the majority of children with bronchiolitis are also treated with antibiotics. The co-existent pneumonia is likely related to the high carriage rate and early acquisition of respiratory pathogens in Indigenous children compared to non-Indigenous children.[2] We have reviewed 100 charts of children hospitalised with bronchiolitis at the RDH and found that the readmission rate of Indigenous children for their next bronchiolitis episode within 6-months was 30% (unpublished). As the majority of these children hospitalised for bronchiolitis are retrieved from remote communities, the impact of the illness and costs plus social dislocation is significant. Furthermore recurrent lower respiratory tract infection is an independent significant risk factor for the later development of bronchiectasis[3] and lower pulmonary function in later years.[4] In addition the severity of the hospitalised episode (oxygen requirement and length of hospital stay) were also risk factors for development of bronchiectasis.[3] In our retrospective chart review we found that Indigenous infants hospitalised with bronchiolitis had more severe bronchiolitis than non-Indigenous infants (unpublished). In Indigenous infants, the severity score at presentation, number of days requiring oxygen and length of hospitalisation were all significantly worse or longer than in non-Indigenous infants (p=0.0001, 0.004, 0.001 respectively). Thus any intervention that is efficacious in reducing the

severity of bronchiolitis and/or readmission for bronchiolitis in Indigenous children would be beneficial in both short and long term outcomes.

3.4.2 Why use azithromycin for bronchiolitis?

There are 2 published randomised controlled trials on RSV-bronchiolitis with contrasting results. In the Turkish study in hospitalised infants, clarithromycin was effective in reducing the length of hospital stay, severity of illness (oxygen use) as well as readmission rate in infants with moderate to severe bronchiolitis.[5] However in the European based study (primarily from Netherlands), azithromycin was not efficacious in infants hospitalised with bronchiolitis.[6] We suspect that while there are many possible reasons for the difference in efficacy, the major reason for the difference found relates to the setting ie infants in Turkey are more likely to have concomitant bacterial infection compared to the affluent European group. Childhood bronchiectasis remains a problem in Turkey[7] similar to that found in Indigenous children living in the NT.[8] It is highly likely that Indigenous infants in our setting will be similar to that of the Turkish infants. However, it is important to establish whether azithromycin is indeed efficacious in our setting given that extrapolating data from developing countries may result in poorer outcomes.[9]

Bronchiolitis in NT Indigenous children is complicated by the known early colonisation of the nasopharynx by bacterial pathogens, notably *Haemophilus influenzae*, *Moraxella catarrhalis* and *Streptococcus pneumoniae*.[2,10] Whilst both are macrolides, azithromycin has several benefits over clarithromycin in the Northern Territory setting. Azithromycin is active against a range of respiratory pathogens which colonise NT indigenous infants from a young age (in particular gram negative bacteria including *H. influenzae*). Also azithromycin is currently widely used in the Northern Territory for bronchiectasis, trachoma and sexually transmitted diseases. It has a longer half life and better tissue penetration than clarithromycin, thus requiring a much shorter treatment regime. Once weekly dosing has been shown to be sufficient for tissue effects lasting over a week.[11] As the pharmacodynamic/kinetic properties of azithromycin are superior to clarithromycin, we have elected to use azithromycin rather then clarithromycin.

Macrolides are a family of antibiotics which, in addition to antibacterial properties, possess potent anti-inflammatory properties and also influence bacterial biofilms.[12] Laboratory studies show that macrolides directly dampen airway inflammation by inhibiting the migration of neutrophils to the respiratory epithelium and inhibits further production of pro-inflammatory cytokines and mediators.[12] Given that acquisition and colonisation of respiratory pathogens occur very early in Indigenous infants (as early as 2 weeks)[2,10] and current data on biofilms present in people with chronic upper respiratory infections, [13] we propose that azithromycin may be a valuable intervention for clinical outcomes of Indigenous infants hospitalised with bronchiolitis.

3.5 Flow chart – Intervention arm

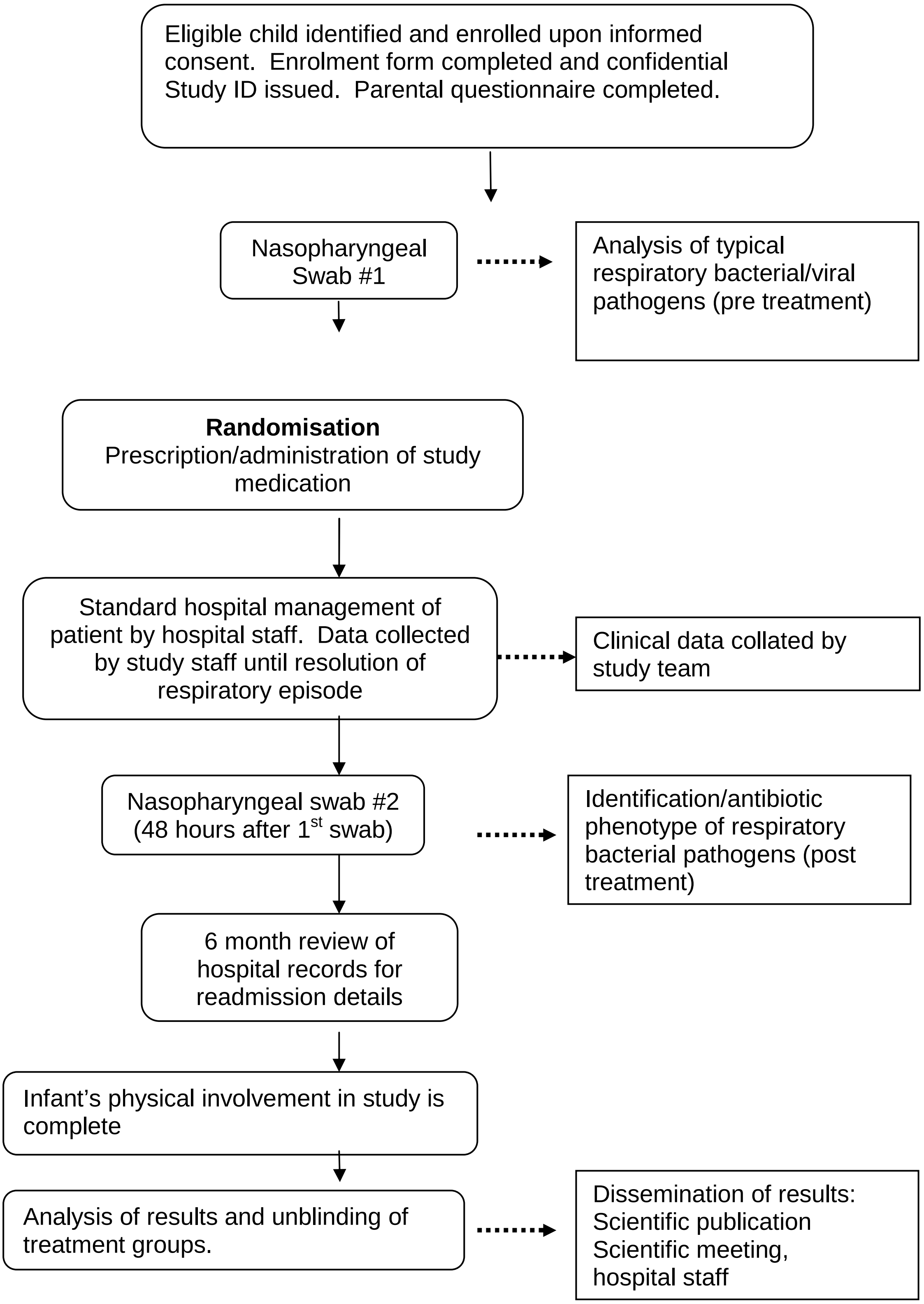

3.6 Study timeline

	2010				2011				2012			
Recruitment/follow up												
Microbiology												
Data analysis, publications, feedback to stakeholders												

4 Study Design

All children admitted to the Paediatric ward RDH & TTH with a diagnosis of bronchiolitis will be identified and recorded on the study log. Eligibility of all children will be determined using the eligibility criteria in 4.1. Children enrolled in this study, eligible children not consented and children who do not meet the eligibility criteria will all be recorded on the study log.

4.1 Eligibility criteria

Inclusion criteria	***Exclusion criteria***
Clinical diagnosis of bronchiolitis	Chronic lung disease
Age 18 months and less	Macrolide contraindication
Requires supplemental oxygen	Administered azithromycin within 7 days
	Concurrent diagnosis of gastroenteritis
	Cyanotic congenital heart disease
	Received O2 longer than 24 hours

4.2 Enrolment protocol

Plain-language educational tools will be used to explain the study to the primary care-giver of a clinically eligible infant. If informed consent is given the infant will be enrolled and randomised to receive either a single, oral dose of azithromycin syrup at 30mg per/kg or an equivalent volume of placebo (syrup).

4.2.1 Enrolment procedure

1. After obtaining informed consent, complete a consent form and give a copy of this and the information sheet to the carer.
2. Complete the enrolment form and the as much of the demographic and medical history as possible from the carer.
3. Use the date of birth and Indigenous status data to determine the treatment strata. There are 4 possible strata
   - **Strata 1** Indigenous 6 months and less
   - **Strata 2** Non Indigenous 6 months and less
   - **Strata 3** Indigenous older than 6 months
   - **Strata 4** Non Indigenous older than 6 months
4. Complete a chart notification sticker and stick in the child's medical chart

4.2.2 Randomisation and blinding of treatment allocation

This is a double-blind, randomised, placebo-controlled trial with concealed allocation. The study medications will be assigned the labels A, B, C or D (two labels assigned to placebo azithromycin and two assigned to azithromycin) by an independent person of the study at Menzies School of Health Research. Neither the study team (researchers, hospital staff) nor parents will know which treatment group (placebo or azithromycin) the infant has been assigned until the results have been analysed. Placebo azithromycin has been specifically manufactured by IDT Australia Limited (Melbourne, Victoria) and has a similar smell and taste to active azithromycin. The manufacture of placebo was

primarily for our NHMRC funded international study on bronchiectasis in Indigenous children.

The treatment allocation list has been computer generated by using stratified block randomisation. There are 4 sheets, each corresponding to the treatment strata. Treatment allocation has been concealed by black sticker. Upon enrolment an infant will be assigned to the next treatment on the appropriate stratified list and the treatment (A, B, C or D) revealed.

Procedure:

1. After identifying the correct strata write the child's name and HRN on the next available line. The number refers to the child's study identification number.
2. Remove the sticker to reveal the study medication allocation (letter A, B, C or D). Record this on the child's chart notification sticker and on the enrolment form.
3. Treating physician to complete ABIS script to order medication/placebo on medication chart (dose 30mg/kg). Medication scripts are found in the study documents and are given to physician by ABIS study staff.
4. ABIS script taken to pharmacy by ABIS study staff and medication dispensed.
5. Take medication back to the paediatric ward and inform the nurse looking after the child that the child has been enrolled in the study.
6. For children enrolled on the weekends, please ensure that scripts are taken down to the Pharmacy before 1000hrs to ensure the pharmacy has enough time to dispense. Administer/supervise administration of first dose of medication.

4.2.3 Clinical samples

4.2.3.1 Nasopharyngeal swabs

a) One nasopharyngeal swab is obtained prior to the administration of the study medication. A second nasopharyngeal swab is obtained 48 hours post administration of study medication. The swabs are stored in separate vials of STGGB medium and stored at -70 degrees prior to dispatch to the Menzies laboratory for processing.

4.3 Study size

We plan to enrol 100 infants total. Children will be stratified by age (6 months and less and over 6 months), Indigenous status (Indigenous and non Indigenous) and site (Darwin and Townsville). The mean length of stay for bronchiolitis at Royal Darwin Hospital is 96 hours. To detect a mean difference of 24 hours in length of stay between treatment groups with an alpha of 0.05 and a power of 90%, the required sample size is 23 infants per group.

4.4 Refusal to participate

Participation in the study is completely voluntary. A participant may choose to refuse to participate in the study. Parents will be reassured that the care of their child will not be compromised in any way by not participating.

4.5 Withdrawal of Participant

Participation in the study is completely voluntary. A participant may choose to withdraw at any stage without giving reason.

4.6 Clinical assessment

Children enrolled in this study will be managed by hospital staff according to standard clinical practice for the management of bronchiolitis. They are not exempt from receiving any care or management protocol specified by the attending physician.

A standardised sheet will be used to collect clinical data. Demographic information and medical history will be obtained from the primary care-giver and from hospital records. The primary and secondary outcome measures (described below) will be monitored from the medical chart until the acute study end-point has been reached (defined as >16 hours off supplemental oxygen and infant is feeding well). Clinical assessment data includes oxygen requirement and level, physiological measurements for clinical severity score (respiratory rate, accessory muscle use, degree of wheeze), other physiological measurement (temperature, heart rate), requirement for other therapies (IV fluids, nutrition, antibiotics). In addition routine investigatory parameters (FBC and chest x-ray findings) will be recorded.

4.7 Microbiology/Virology

4.7.1 Bacteriology of nasopharyngeal swabs

Swabs for microbiological analysis are stored in STGGB at -70 (in accordance with WHO guidelines). Presence of *Haemophilus influenzae*, *Moraxella catarrhalis* and *Streptococcus pneumoniae* will be identified using culture methods established in the Menzies laboratory. The antibiotic resistance phenotype will also be determined pre and post study medication administration.

Additionally, nucleic acids will be extracted from 0.2 ml of each nasopharyngeal swab specimen using the High Pure Viral Nucleic Acid kit (Roche Diagnostics, Australia), according to the manufacturer's instructions. Monospecific PCR and reverse transcriptase PCR (RT-PCR) method will be used to detect *Mycoplasma pneumoniae*, *Chlamydia pneumoniae* [14], coronaviruses [15], bocavirus [16] and human metapneumovirus (hMPV) [17,18, 19] whereas multiplex PCR and RT-PCR will be used to detect adenovirus, parainfluenza (1, 2 3), influenza (A and B), and respiratory syncytial virus (RSV) [20,21]. These methods have been previously validated in the QLD Paediatric Infectious Disease Laboratory.

4.7.2 Respiratory pathogens in nasopharyngeal aspirates

NPA collected in the standard clinical manner will be frozen at -80℃. Upon thawing nucleic acids will be extracted from 0.2 ml of each NPA specimen using the High Pure Viral Nucleic Acid kit (Roche Diagnostics, Australia), according to the manufacturer's instructions. Monospecific PCR and reverse transcriptase PCR (RT-PCR) method will be used to detect *Mycoplasma pneumoniae*, *Chlamydia pneumoniae* [14], coronaviruses [15], bocavirus [16] and human metapneumovirus (hMPV) [17,18, 19] whereas multiplex PCR and RT-PCR will be used to detect adenovirus, parainfluenza (1, 2 3), influenza (A and B), and respiratory syncytial virus (RSV) [20,21]. These methods have been previously validated in the QLD Paediatric Infectious Disease Laboratory.

4.8 Data analysis

4.8.1 Primary outcome: length of stay for bronchiolitis episode. The primary outcome (length of hospital stay) will be compared between infants receiving placebo or azithromycin using non-parametric analysis. Data within the Indigenous status will be analysed separately.

4.8.2 Secondary outcome: Length of time on supplemental oxygen; readmission to hospital within 6 months of this episode. All clinical secondary outcomes and laboratory data will also be analysed using non-parametric analysis. Categorical data will be compared using Chi square (or Fishers Exact).

5 Data storage and record keeping

All information collected from study participants will be treated as strictly confidential and accessibly by study personnel only. Data will be stored in a restricted-access building at TTH and then sent to Menzies School of Health Research in a locked filing cabinet or password-protected electronic database. Study participants will be issued with a numerical code and will not be identifiable in any publications. Data will be stored for 15 years or until the child reaches the age of 25 (whichever is greater).

Samples to be used for research purposes (nasopharyngeal swab and nasopharyngeal aspirate) will be processed and stored in the restricted-access PC2 laboratory of Menzies School of Health Research by trained study personnel. Samples sent to the QPID laboratory for analysis will be transported by courier and will be identified by numerical code only.

6 Quality control/quality assurance

This study will be conducted in accordance with the NHMRC Guidelines on Ethical Matters in Aboriginal and Torres Strait Islander Health Research, the Note for Guidance on Good Clinical Practice and the Declaration of Helsinki. Ethics approval and Clinical Trial Protocol committee reviews for the protocols and subsequent amendments will be sought prior to implementation.

Adequately trained and experienced personnel will be employed for trial related activities. This will be documented by way Curriculum Vitae to be kept at Menzies School of Health Research.

6.1 Safety parameters

Safety profile, as measured by the incidence, intensity, and relationship to study drug of adverse events and serious adverse events.

An **adverse event** is any untoward medical occurrence experienced by the participant while undergoing treatment in a clinical trial, and up to one week after the dose of Study medicine. Adverse events include temporary, moderate discomfort and disability to the participant.

A **serious adverse event** is defined as any event that is fatal or life threatening is permanently disabling to the participant, any admission to hospital, or an overdose that requires additional medical management

6.2 Methods of assessing safety parameters

Side effects of Azithromycin are rare. Those listed in MIMS Annual 2001 included: nausea, vomiting, diarrhoea, or abdominal pain. Rare but potentially serious side effects listed are angioedema and cholestatic jaundice.

The children enrolled in the study have been admitted to TTH and RDH and as such are monitored according to hospital protocol. The child’s progress is monitored daily by the study staff via the medical chart and the Nursing Unit Manager.

6.3 Flowchart – adverse event reporting

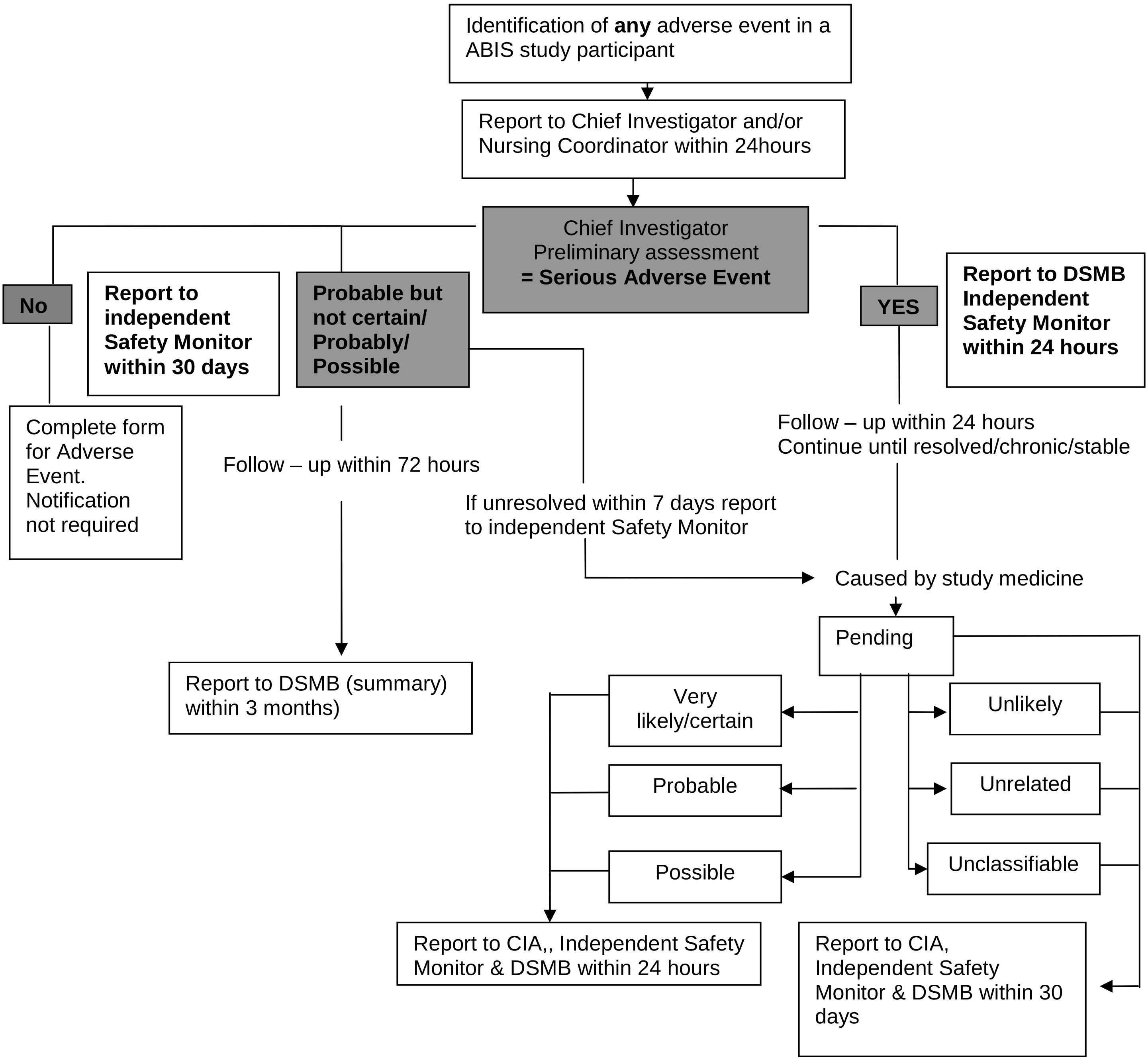

6.4 Classification and reporting of serious adverse events

All SAEs will be investigated and assessed to determine whether the event was caused by administration of a study medicine. Causality will be assessed against the definitions below, which are based on standard WHO criteria.

Classification	Definition	Report to Safety Monitor
**Very likely/ Certain**	A clinical event with a plausible time relationship to medication administration/non-administration and which cannot be explained by concurrent disease or other drugs or chemicals	**Within 24 hours**
**Probable**	A clinical event with a reasonable time relationship to medicine administration/non-administration; is unlikely to be attributed to concurrent disease or other drugs or chemicals	**Within 24 hours**
**Possible**	A clinical event with a reasonable time relationship to medicine administration/non-administration, but which could also be explained by concurrent disease or other drugs or chemicals	**Within 24 hours**
**Unlikely**	A clinical event whose time relationship to medicine administration/non- administration makes a causal connection improbable, but which could plausibly be explained by underlying disease or other drugs or chemicals	**Within 30 days**
**Unrelated**	A clinical event with an incompatible time relationship and which could be explained by underlying disease or other drugs or chemicals	**Within 30 days**
**Unclassifiable**	A clinical event with insufficient information to permit assessment and identification of the cause	**Within 30 days**

All SAEs will be investigated by the Chief Investigator in consultation with the Independent Safety Monitor. Causality will be assigned using the above definitions. The findings of each investigation will be reported to the Independent Safety Monitor within the time periods specified above in a standard format (see SAE Brief Case Summary).

SAEs with a causality classification of “very likely/certain”, “probable” or “possible” must also be reported to the Chief Investigator and Chair of the DSMB within 24 hours of the determination. The reports will consist of line listed data showing participant identification number, community, randomisation date, age, group allocation, diagnosis of SAE, causality of SAE, outcome of SAE, continuation/withdrawal from study. Other causality classifications will be reported to the DSMB in summary format within 30 days.

6.5 DSMB (Data Safety Monitoring Board)

The DSMB is an independent group of experts that advises the study investigators. The members of the DSMB serve in an individual capacity and provide their expertise and recommendations to guide the clinical trial. An elected member of the DSMB will become the independent safety monitor of the study.

The DSMB, unblinded, will review all events (i.e. mild, moderate, severe or life-threatening events), whether they are related or unrelated to the administration of study drug. The DSMB will meet in person or via telephone link up at the start of the trial and on a six-monthly basis after that. The DSMB will also review any Serious Adverse Event in an expeditious manner as they are reported.

6.6 DSMB Independent safety monitor

A voting member of the DSMB, this position is the primary DSMB contact for serious adverse event reporting

Liases with the Chief Investigator and health care provider to ensure best standard of care is provided for participant.

Reviews the classification of SAEs and directs further investigations as necessary.

Provides advice on further information required to assign causality.

7 Publication policy

Publication and reporting of results and outcomes of this trial will be in accordance with Menzies Publication Policy. Publication of results will be subjected to fair peer-review. Authorship will be given to all persons providing significant input into the conception, design, and execution or reporting of the research according to Menzies Statement of Record Integrity.

No person who is an author, consistent with this definition, will be excluded as an author without their permission in writing. Authorship will be discussed between researchers prior to study commencement (or as soon as possible thereafter) and reviewed whenever there are changes in participation. All conflicts arising through disputes about authorship will be reviewed by the Menzies director. Acknowledgement will be given to collaborating institutions and hospitals and other individuals and organizations providing finance or facilities.

Annual reports will be submitted to the appropriate Human Research Ethics Committee.

Regular study progress reports will be made available to the clinical and nursing staff of Royal Darwin Hospital and TTH

A final report will be made available to all stakeholders including Royal Darwin Hospital, Menzies School of Health Research, funding bodies and the HREC. No study participant will be individually identifiable in any publication, written or oral.

8 Ethics

This project received clearance by the Human Research Ethics Committee of the Northern Territory Department of Health and Families and Menzies School of Health Research; 07/60 and the Townsville Health Service District Human Research Ethics Committee HREC/10/QTHS/9.

Infants to 18 months of age will be involved in the study. The study will be explained verbally to the child's carer based on the information sheet and consent flipchart. Parents/guardians will sign a consent form.

The carer of eligible children will be fully informed about the study, the data collection, procedures and possible risks involved in taking the study medication (azithromycin or

placebo). Nasopharyngeal swab will be collected by a trained member of the study team. The nasopharyngeal aspirate is performed as part of hospital management for bronchiolitis and is performed by hospital staff. Azithromycin is currently being used in the Northern Territory in children with chronic suppurative lung disease.

9 Registering of trial

This trial was registered with the Australian and New Zealand Clinical Trials register on the 28 March 2008 (ACTRN12608000150347).

10 References

1 Bolisetty S, Wheaton G, Chang AB. Respiratory syncytial virus infection and immunoprophylaxis for selected high-risk children in Central Australia. *Aust J Rural Health* 2005; 13**:** 265-270.

2. Stubbs E, Hare K, Wilson C, Morris P, Leach AJ. Streptococcus pneumoniae and noncapsular Haemophilus influenzae nasal carriage and hand contamination in children: a comparison of two populations at risk of otitis media. *Pediatr Infect Dis J* 2005; 24**:** 423-428.
3. Valery PC, Torzillo PJ, Mulholland EK, Boyce NC, Purdie DM, Chang AB. A hospital-based case-control study of bronchiectasis in Indigenous children in Central Australia. *Pediatr Infect Dis J* 2004; 23**:** 902-908.
4. Tennant PWG, Gibson JG, Pearce MS. Lifecourse predictors of adult respiratory function: results from the Newcastle Thousand Families Study. *Thorax* 2008; epub ahead:11 April 2008.
5. Tahan F, Ozcan A, Koc N. Clarithromycin in the treatment of RSV bronchiolitis: a double-blind, randomised, placebo-controlled trial. *Eur Respir J* 2007; 29**:** 91-97.
6. Kneyber MC, van Woensel JB, Uijtendaal E, Uiterwaal CS, Kimpen JL. Azithromycin does not improve disease course in hospitalized infants with respiratory syncytial virus (RSV) lower respiratory tract disease: A randomized equivalence trial. *Pediatr Pulmonol* 2008; 43**:** 142-149.
7. Karadag B, Karakoc F, Ersu R, Kut A, Bakac S, Dagli E. Non-cystic-fibrosis bronchiectasis in children: a persisting problem in developing countries. *Respiration* 2005; 72**:** 233-238.
8. Chang AB, Grimwood K, Mulholland EK, Torzillo PJ. Bronchiectasis in Indigenous children in remote Australian communities. A position statement. *Med J Aust* 2002; 177**:** 200-204.
9. Chang AB, Torzillo PJ, Boyce NC, White AV, Stewart PA, Wheaton GR, Purdie DM, Wakerman J, Valery PC. Zinc and Vitamin-A supplementation in Indigenous children hospitalised with episodes of lower respiratory tract infection: a randomised controlled trial. *Med J Aust* 2006; 184**:** 107-112.
10. Leach AJ, Boswell JB, Asche V, Nienhuys TG, Mathews JD. Bacterial colonization of the nasopharynx predicts very early onset and persistence of otitis media in Australian aboriginal infants. *Pediatr Infect Dis J* 1994; 13**:** 983-989.
11. Blasi F, Cazzola M, Tarsia P, Cosentini R, Aliberti S, Santus P, Allegra L. Azithromycin and lower respiratory tract infections. *Expert Opin Pharmacother* 2005; 6**:** 2335-2351.
12. Giamarellos-Bourboulis EJ. Macrolides beyond the conventional antimicrobials: a class of potent immunomodulators. *Int J Antimicrob Agents* 2008; 31**:** 12-20.
13. Galli J, Ardito F, Calo L, Mancinelli L, Imperiali M, Parrilla C, Picciotti PM, Fadda G. Recurrent upper airway infections and bacterial biofilms. *J Laryngol Otol* 2007; 121**:** 341-344.
14. Didierlaurent A, Goulding J, Patel S, Snelgrove R, Low L, Bebien M, Lawrence T, van Rijt LS, Lambrecht BN, Sirard JC, Hussell T. Sustained desensitization to bacterial Toll-like receptor ligands after resolution of respiratory influenza infection. *J Exp Med* 2008; -Jan 28; [Epub ahead of print].
15. Chang AB, Cox NC, Purcell J, Marchant JM, Lewindon PJ, Cleghorn GJ, Ee LC, Withers GD, Patrick MK, Faoagali J. Airway cellularity, lipid laden macrophages and microbiology of gastric juice and airways in children with reflux oesophagitis. *Respir Res* 2005; 6**:** 72.
16. McDonough EA, Barrozo CP, Russell KL, Metzgar D. A multiplex PCR for detection of Mycoplasma pneumoniae, Chlamydophila pneumoniae, Legionella pneumophila, and Bordetella pertussis in clinical specimens. *Mol Cell Probes* 2005; 19**:** 314-322.
17. Arden KE, McErlean P, Nissen MD, Sloots TP, Mackay IM. Frequent detection of human rhinoviruses, paramyxoviruses, coronaviruses, and bocavirus during acute respiratory tract infections. *J Med Virol* 2006; 78**:** 1232-1240.

18. Sloots TP, McErlean P, Speicher DJ, Arden KE, Nissen MD, Mackay IM. Evidence of human coronavirus HKU1 and human bocavirus in Australian children. *J Clin Virol* 2006; 35**:** 99-102.
19. Mackay IM, Jacob KC, Woolhouse D, Waller K, Syrmis MW, Whiley DM, Siebert DJ, Nissen M, Sloots TP. Molecular assays for detection of human metapneumovirus. *J Clin Microbiol* 2003; 41**:** 100-105.
20. Maertzdorf J, Wang CK, Brown JB, Quinto JD, Chu M, de GM, van den Hoogen BG, Spaete R, Osterhaus AD, Fouchier RA. Real-time reverse transcriptase PCR assay for detection of human metapneumoviruses from all known genetic lineages. *J Clin Microbiol* 2004; 42**:** 981-986.
21 Syrmis MW, Whiley DM, Thomas M, Mackay IM, Williamson J, Siebert DJ, Nissen MD, Sloots TP. A sensitive, specific, and cost-effective multiplex reverse transcriptase-PCR assay for the detection of seven common respiratory viruses in respiratory samples. *J Mol Diagn* 2004; 6**:** 125-131.
